# Coffinite formation from UO_2+x_

**DOI:** 10.1038/s41598-020-69161-1

**Published:** 2020-07-22

**Authors:** Stéphanie Szenknect, Delhia Alby, Marta López García, Chenxu Wang, Renaud Podor, Frédéric Miserque, Adel Mesbah, Lara Duro, Lena Zetterström Evins, Nicolas Dacheux, Jordi Bruno, Rodney C. Ewing

**Affiliations:** 1 ICSM, Univ Montpellier, CEA, CNRS, ENSCM, 30207 Bagnols sur Cèze, France; 2 Amphos 21, Consulting, Carrer Veneçuela, 103, Planta 2, 08019 Barcelona, Spain; 3 Swedish Nuclear Fuel and Waste Management Co, Blekholmstorget 30, 101 24 Stockholm, Sweden; 4 Department of Geological Sciences, Stanford University, Stanford, CA 94305-2115 USA; 5 DES-Service de la Corrosion et du Comportement des matériaux dans leur Environnement (SCCME), CEA, Université Paris-Saclay, 91191 Gif-Sur-Yvette, France

**Keywords:** Environmental sciences, Environmental chemistry, Inorganic chemistry, Nuclear chemistry

## Abstract

Most of the highly radioactive spent nuclear fuel (SNF) around the world is destined for final disposal in deep-mined geological repositories. At the end of the fuel’s useful life in a reactor, about 96% of the SNF is still UO_2_. Thus, the behaviour of UO_2_ in SNF must be understood and evaluated under the weathering conditions of geologic disposal, which extend to periods of hundreds of thousands of years. There is ample evidence from nature that many uranium deposits have experienced conditions for which the formation of coffinite, USiO_4_, has been favoured over uraninite, UO_2+x_, during subsequent alteration events. Thus, coffinite is an important alteration product of the UO_2_ in SNF. Here, we present the first evidence of the formation of coffinite on the surface of UO_2_ at the time scale of laboratory experiments in a solution saturated with respect to amorphous silica at pH = 9, room temperature and under anoxic conditions.

## Introduction

Uraninite, UO_2+x_ is the most common U^4+^ mineral in nature followed by coffinite, USiO_4_, which is found as a primary phase or an alteration product in many uranium deposits. Coffinite, tetragonal, is isostructural with zircon (ZrSiO_4_) and thorite (ThSiO_4_); however, coffinite can contain some water either as H_2_O or OH groups^1^. Altered uraninite and coffinite have been documented from Oklo, Gabon^2–5^, deposits in the Athabasca Basin^4,6,7^ and Elliot Lake, Canada^8^. Other examples include Jachymov, Czech Republic^9^ or La Crouzille district, France^10^. For many years, coffinite had gone unrecognized in most uranium deposits, particularly uranium roll-front deposits, as a distinct phase because of its fine grain size and intimate association with uraninite^5,6,11,12^. The alteration of uraninite to coffinite is a key event for UO_2_ in nature and UO_2_ in spent fuel in a geologic repository. Coffinite, being a U^4+^-silicate, is associated with reducing environments, with sulphides and organic matter^1^, where it likely precipitated from neutral to weakly alkaline fluids. Coffinite formation in sedimentary uranium deposits is associated with relatively low temperatures, 80–130 °C. A detailed investigation of meteoric roll-front deposits in the Athabasca basin, suggest an estimated temperature of coffinite precipitation in the uranium front of no greater than 50 °C^13^. Even though laboratory experiments report coffinite formation at 150–250 °C^14,15^, it appears that these elevated temperatures are not required to form coffinite in nature.

Although coffinite is abundant in uranium ore deposits, its synthesis has been a major challenge since its initial description as a mineral in 1955^16^. A number of investigators have sought to obtain pure synthetic coffinite, but only a few have succeeded^14,15,17–21^. Synthetic coffinite was always obtained under hydrothermal conditions. Systematically, the samples obtained were a mixture of phases, mainly composed of fine grains of USiO_4_, nanoparticles of UO_2_ and amorphous SiO_2_. All of these attempts to synthesize coffinite indicate that there is only a narrow range in terms of temperature, pH, uranium and silicate ions concentrations and oxygen fugacity for which the formation of coffinite over UO_2_ is favored. More recently, the determination of thermodynamic data has been made possible thanks to the preparation of a single-phase USiO_4_ sample^22,23^. These data confirm the relative stability of coffinite and UO_2_ as a function of groundwater composition. Thermodynamic calculations indicate unambiguously that coffinite is less stable than the quartz and UO_2_ (cr) mixture at 25 °C. However, coffinite precipitates in solutions undersaturated with respect to amorphous UO_2_⋅2H_2_O (am) in silicate solutions with concentrations typical of groundwater (i.e.[Si]_tot_ between 7 × 10^–5^ and 5 × 10^–3^ mol L^−1^)^24^. This result supports the idea that the uraninite-coffinite transformation requires a prior destabilization of uraninite and that this could be caused by self-irradiation, leading to metamictization of the solid and radiolysis of water and/or surface oxidation at moderate oxygen fugacities. Non-stoichiometry is also common in natural uraninite and could have a significant effect on uraninite reactivity and solubility^9^. Coffinite could thus be preferentially formed at the interface between UO_2+x_ resulting from the oxidation of UO_2_ surface layer and the silicate-bearing fluids.

Similar to natural uraninite, recent findings regarding the thermodynamic stability of coffinite have renewed the interest in considering coffinite as a potential alteration product of SNF in a geologic repository, particularly under reducing conditions. During in-reactor irradiation UO_2_ fuel pellets experience many chemical modifications and considerable radiation-induced defect formation. Such microstructural changes in UO_2_ matrix occur from the nanometer up to the macroscopic scale^25^ and, by similar to uraninite, could enhance the possibility of the formation of coffinite^26^. Most of the geologic sites under investigation for underground repositories are located in undisturbed clay-rich rock or granite, with silica-rich groundwaters ([Si]_tot_ ~ 10^–4^ mol/L), deep enough to have reducing conditions (typical Eh range from − 50 to − 300 mV)^27,28^. Understanding the interaction of used fuel with the silicate-rich groundwaters is critical to evaluate the safety of different disposal strategies, as the coffinitization process has not been considered until now. In this paper:

- We show for the first time, at laboratory time scale, the formation of coffinite from UO_2_ in the presence of solution saturated with respect to SiO_2_(am) under conditions typical of near-surface uranium deposits and deep-mined geologic repositories for SNF.
- We have constrained the conditions of formation in an Eh–pH diagram where the precipitation of coffinite is favoured over UO_2_⋅2H_2_O (am).
- We show that coffinite precipitation could lower the uranium release from the UO_2_ matrix of SNF through oxidative weathering in the presence of oxygen in the geological repository.
- Dissolution assisted by silicate ions and precipitation under slightly oxidative conditions (i.e., Eh between -100 and + 100 mV) explains the coexistence of uraninite and coffinite in uranium ore deposits.

## Experimental results

UO_2_ powder was synthesized, then sintered under reducing conditions to maintain uranium in the tetravalent oxidation state. UO_2_ individual pellet was characterized by X-ray diffraction (XRD), scanning electron microscopy (SEM) and X-ray photoelectron spectroscopy (XPS). Details of the synthesis and characterization are included in the “Experimental methods” section.

**Figure 1:**
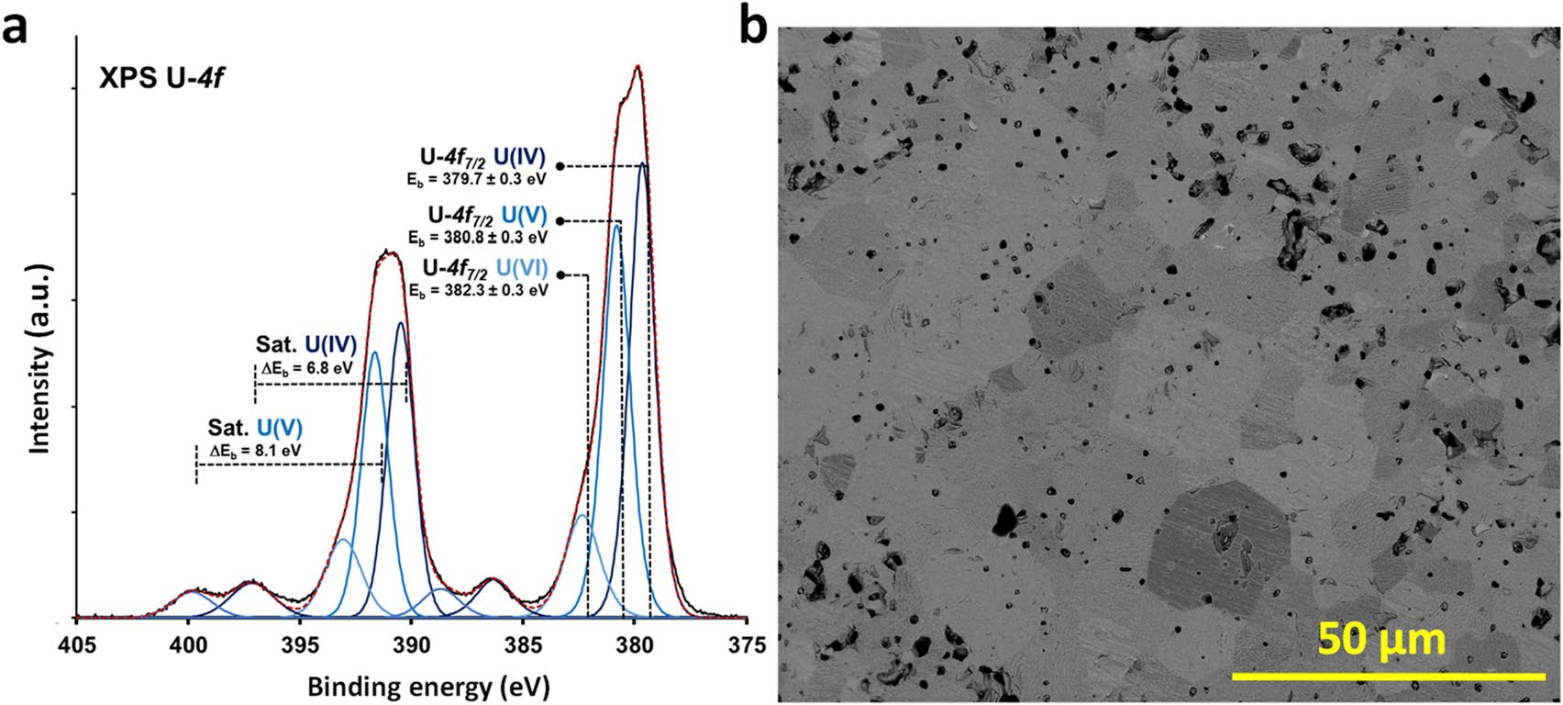
(**a**) U-4f core levels XPS spectrum of UO_2_ pellet before leaching. (**b**) SEM micrograph (BSE mode) of the surface of the UO_2_ pellet before leaching experiment. Scale bar 50 µm.

For the pellet treated at high temperature under vacuum, the value of the unit cell parameter obtained by Rietveld refinement was: a = 546.95(1) pm (Fig. S1 of the supporting information). This value was compared with the unit cell parameter determined by Leinders et al.^29^ for stoichiometric UO_2_ (a = 547.127 (8) pm). This indicates that the sintered pellet has not oxidized to UO_2+x_. However, three main contributions were needed to fit the experimental U-4f_7/2_ core level XPS spectrum of UO_2_ pellet (Fig. 1a). These contributions were attributed to U^4+^, U^5+^ and U^6+^ oxidation states with U-4f_7/2_ peak binding energies of 379.7 ± 0.3 eV; 380.8 ± 0.3 eV and 382.3 ± 0.3 eV, respectively^14,30^. The presence of shake-up satellite peaks at 6.8 eV and 8.1 eV from the main U-4f_7/2_ peaks showed that uranium oxidation states were mainly U^4+^ and U^5+^. In situ Ar^+^ ion etching led to U-4f core levels spectrum with only one U^4+^ contribution. This confirmed that uranium in the bulk material was U^4+^, while U^5+^ and U^6+^ were only present as a thin oxidation layer at the pellet surface. SEM images of the pellet before leaching (Fig. 1b) showed large grains of 10–25 µm in size. Grain “pull-out” was also observed and attributed to the polishing step. This grain pull-out contributed to the significant increase of the open porosity in the pellet, and thus an increased reactive surface area.

**Figure 2:**
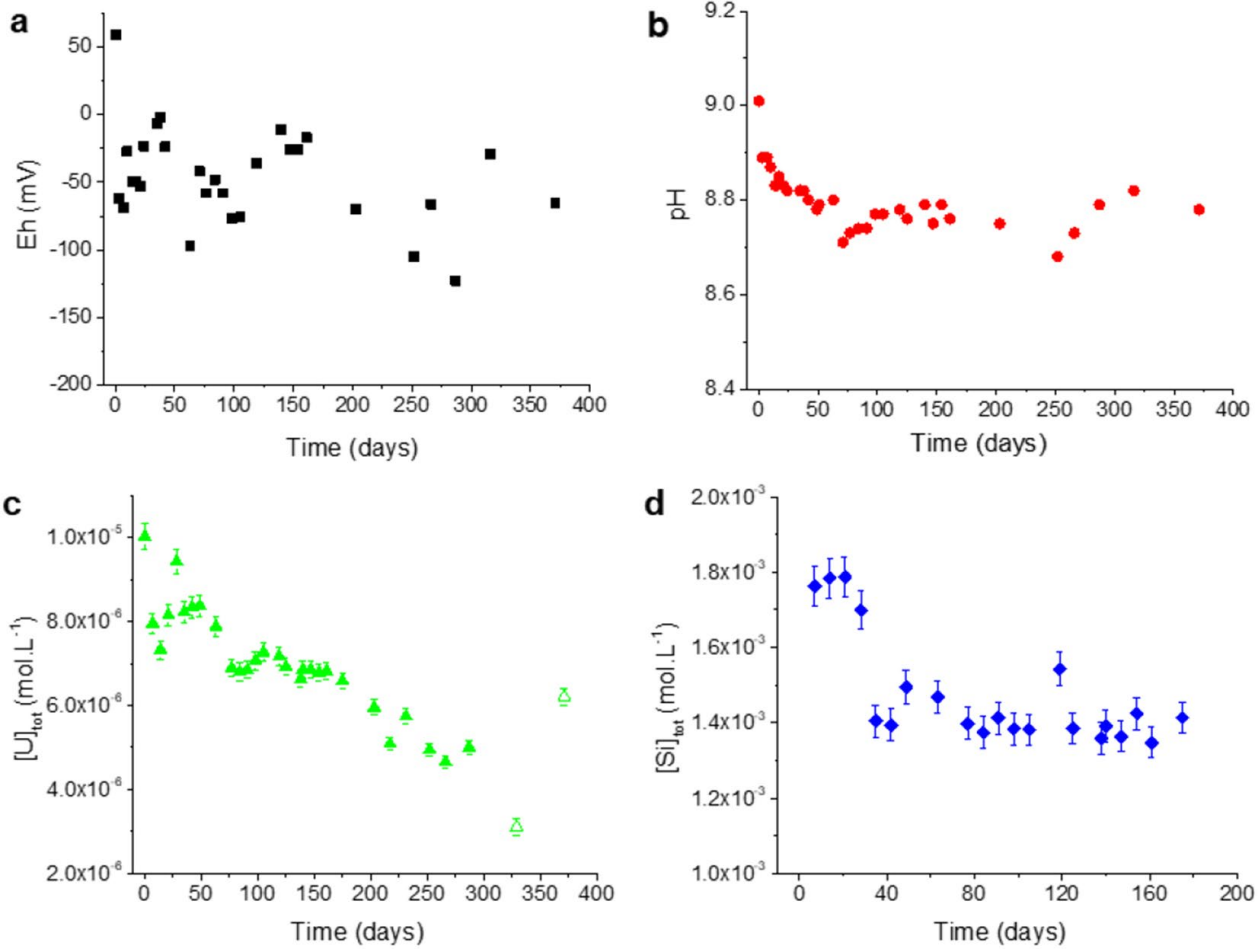
Eh (**a**); pH (**b**); uranium (**c**) and silicate (**d**) elemental concentrations during the leaching of the UO_2_ pellet (open symbols represent data obtained after ultrafiltration of the solution).

This pellet was leached at room temperature, under anoxic conditions (pO_2_ ≤ 1 ppm), with a solution slightly undersaturated with respect to amorphous silica at 25 °C and at pH = 8.76 (i.e.[Si]_tot_ = (1.77 ± 0.03) × 10^–3^ mol L^−1^), but oversaturated with respect to USiO_4_ coffinite (i.e.[U]_tot_ = 10^–5^ mol L^−1^). pH, Eh, Si and U elemental concentrations were monitored during the leaching experiment (Fig. 2).

**Figure 3:**
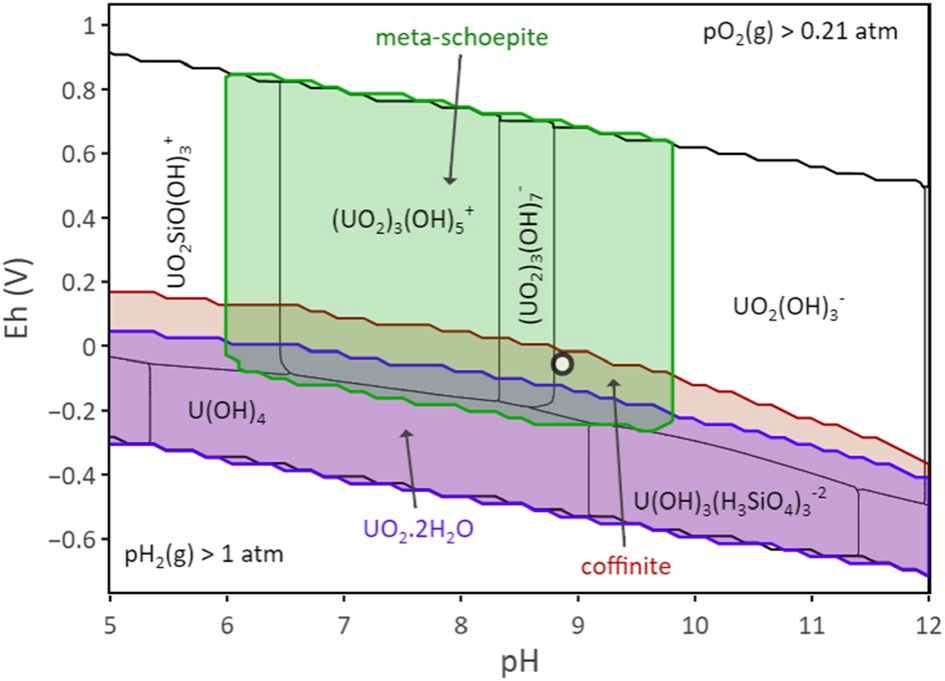
Pourbaix diagram for U. Predominance domains of the major aqueous species and solid phases are shown as a function of the reduction potential, Eh(V) and pH for total U, [U]_tot_ = 5 × 10^–6^ mol L^−1^ in water containing silicate ions, [Si]_tot_ = 2 × 10^–3^ mol L^−1^ and in equilibrium with the atmosphere. Calculations were made considering coffinite stability domain proposed by Szenknect et al.^22^ and the formation constant of the hydroxosilicate complex, U(OH)_3_(H_3_SiO_4_)_3_^2−^ proposed by Mesbah et al.^15^ UO_2_(cr) is not allowed to be present in the calculations. Symbol correspond to experimental data at equilibrium (≥ 100 days). Calculations performed by using the Thermochimie database (https://www.thermochimie-tdb.com).

The results in Fig. 2 indicate that the conditions stabilized after 100 days of contact with the following average and standard deviation values: pH = 8.76 ± 0.03; Eh = − 55 ± 35 mV; [Si]_tot_ = (1.41 ± 0.05) × 10^–3^ mol L^−1^. However, the uranium elemental concentration decreased regularly in solution until 350 days of contact with solution. Experimental data obtained at steady state are plotted together within the predominance diagram of the uranium system (Fig. 3). Compared to the literature, the introduction of silicate ions in solution strongly modifies the usual predominance domains of the major uranium aqueous species and minerals^31^. As can be seen in this diagram, experimental data fall within the stability range of U^6+^ species, UO_2_(OH)_3_^−^ and (UO_2_)_3_(OH)_7_^−^. Regarding the solid phases, experimental data fall in the stability domain of coffinite, which indicates that anoxic conditions led to Eh values of an appropriate range to form coffinite. Examination of the Pourbaix diagram for U (Fig. 3) shows a narrow domain in Eh values where coffinite can be formed coexisting with meta-schoepite, UO_3_⋅0.9H_2_O under slight reducing conditions, UO_2_⋅2H_2_O (am) being stable under more reducing potentials.

The solid/solution interface was characterized by Environmental-SEM (ESEM) and Grazing Incidence-XRD (GI-XRD). Finally, the experiment was stopped after 371 days and the UO_2_ pellet was withdrawn from the solution for characterization by High Resolution Transmission Electron Microscopy (HR-TEM).

**Figure 4:**
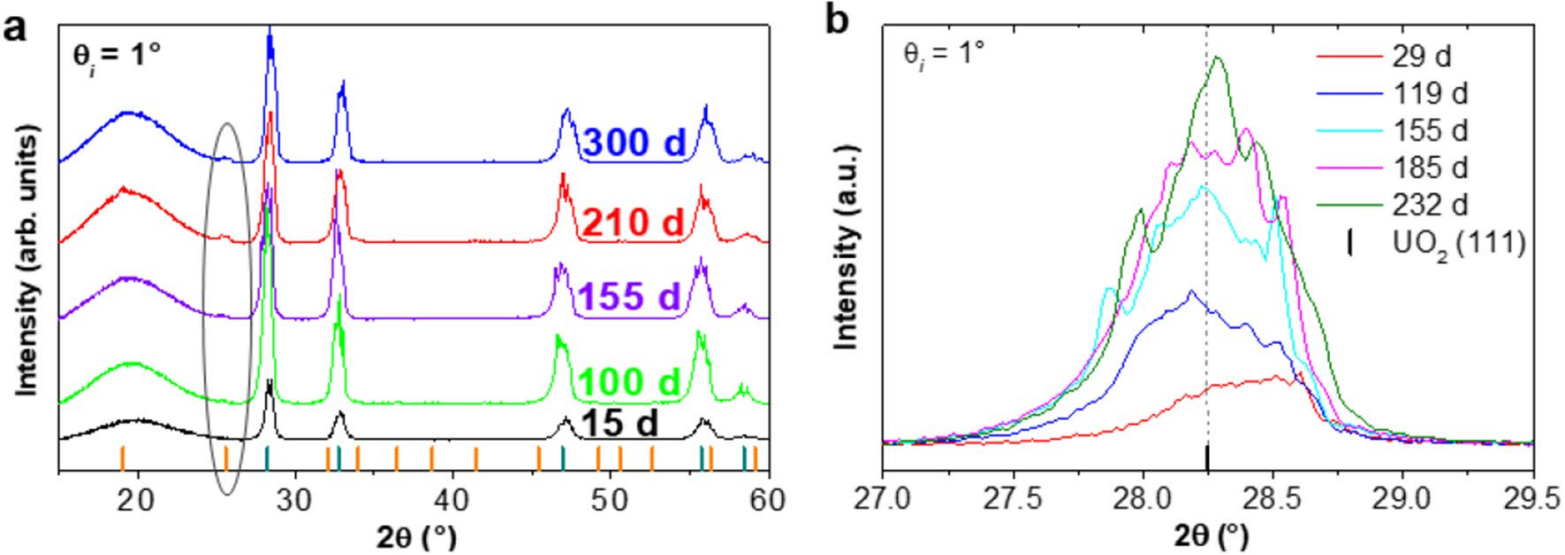
(**a**) GI-XRD patterns obtained at θ_i_ = 1° for different leaching times (in days). The Bragg peak positions characteristic of UO_2_ (PDF: 00-005-0550) and USiO_4_ (PDF: 00-011-0420) are shown with black and orange bars, respectively. (**b**) Extract of the (111) diffraction peak of UO_2_.

During exposure to the silicate solution, GI-XRD patterns were collected for an incident angle, θ_*i*_ of 1° at various times and checked for the appearance of new peaks that would indicate the formation of coffinite at the surface of the UO_2_ pellet. The X-ray penetration depth in UO_2_ at this grazing incident angle was estimated to be 120 nm by Tracy et al.^32^ GI-XRD diffractograms (Fig. 4a) showed the appearance of peaks characteristic of the tetragonal (*I*4_1_/*amd*) coffinite phase after 155 days of solution contact. The presence of the coffinite peaks became obvious after 210 days, whereas patterns obtained showed no evidence of meta-schoepite formation. Particular analysis of the (111) diffraction maximum of UO_2_ (Fig. 4b) suggests the evolution of the fluorite-structure. Patterns obtained showed a shift of the positions of the XRD lines to smaller 2θ angles, corresponding to larger unit-cell parameters. With time, asymmetric peak broadening decreased. Qualitatively, these results indicated an increase of unit cell volume, caused by the decreasing contribution of the oxidized surface layer. This UO_2+x_ layer being more soluble than stoichiometric UO_2_, it most likely has preferentially dissolved at the beginning of the dissolution experiments^33^. Nevertheless, U concentration in solution decreased. This result shows that under the experimental conditions investigated, available U is removed from solution. The solution was initially spiked with U to reach conditions oversaturated with respect to coffinite. The precipitation of coffinite would explain the decrease of Si and U concentration in the bulk solution. This mechanism could also trigger the dissolution of the UO_2+x_ layer by creating undersaturated conditions for UO_2+x_ at the pellet/solution interface.

**Figure 5:**
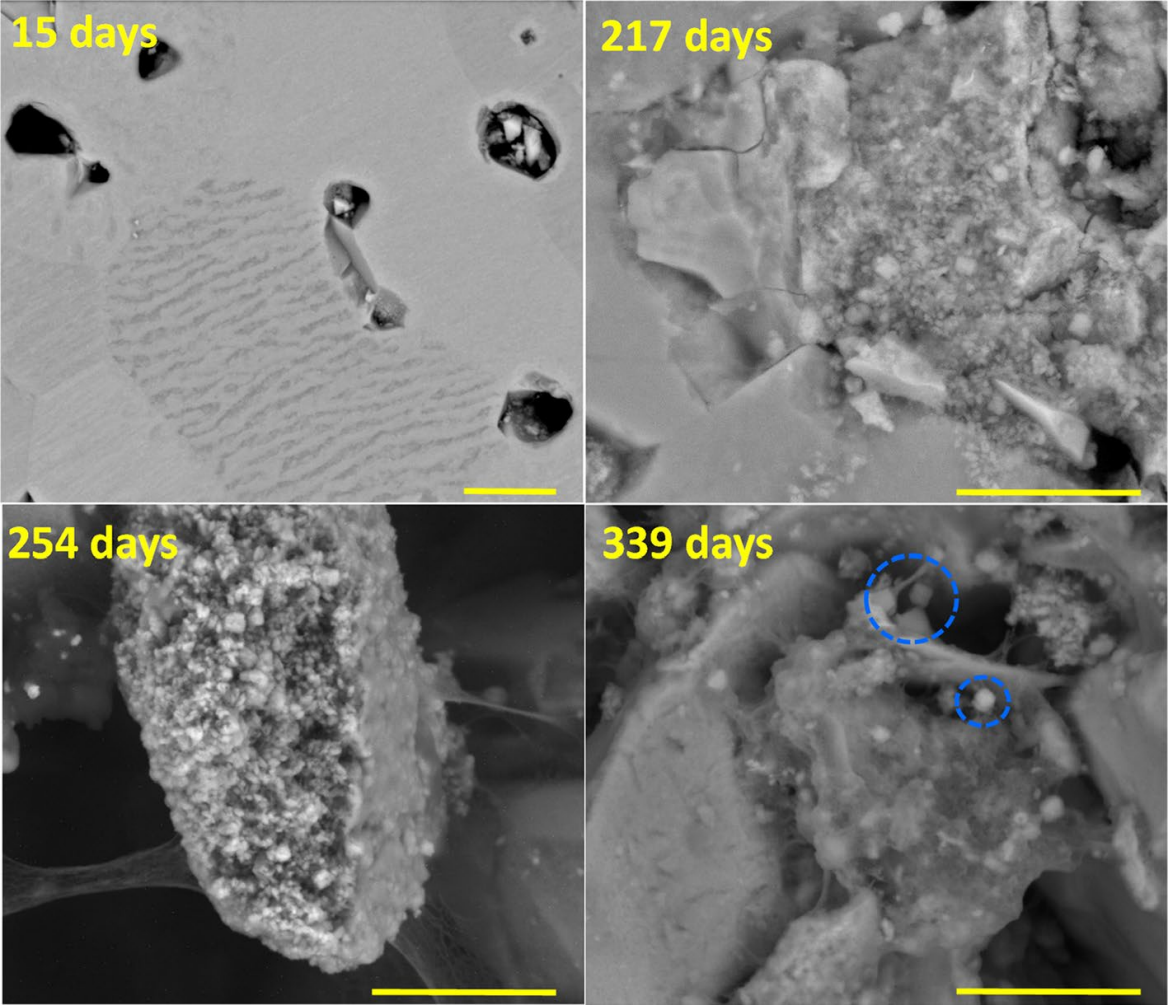
SEM micrographs (BSE mode) of the UO_2_ pellet recorded at different leaching times and high magnification. The blue dotted circles indicate neoformed grains with bipyramidal morphology characteristic of zircon-type crystals. Scale bars 2 µm.

The surface of the UO_2_ pellet was observed regularly by ESEM. Selected micrographs recorded at high magnification highlight the evolution of the UO_2_ grains at the solid/solution interface (Fig. 5). Selected micrographs recorded at low magnification are presented in Fig. S3 of the supporting data to illustrate massive grain detachment at the pellet surface. These results showed that silicate ions had a deleterious effect on the UO_2_ pellet microstructure. As a consequence, the surface area of the pellet in contact with the solution increased through the development of grains roughness and numerous cavities.

After a short contact time with the silicate solution, grain detachment was observed (Fig. 5 at 15 days), the surface of the some grains was altered and small particles were observed in pores. After longer contact times and the appearance of XRD lines associated to coffinite on the GI-XRD patterns (Fig. 5 at 217; 254 and 339 days), the surface of altered grains was covered by small particles embedded in a gel. Some of these particles exhibited a bipyramidal morphology characteristic of coffinite^15^.

**Figure 6:**
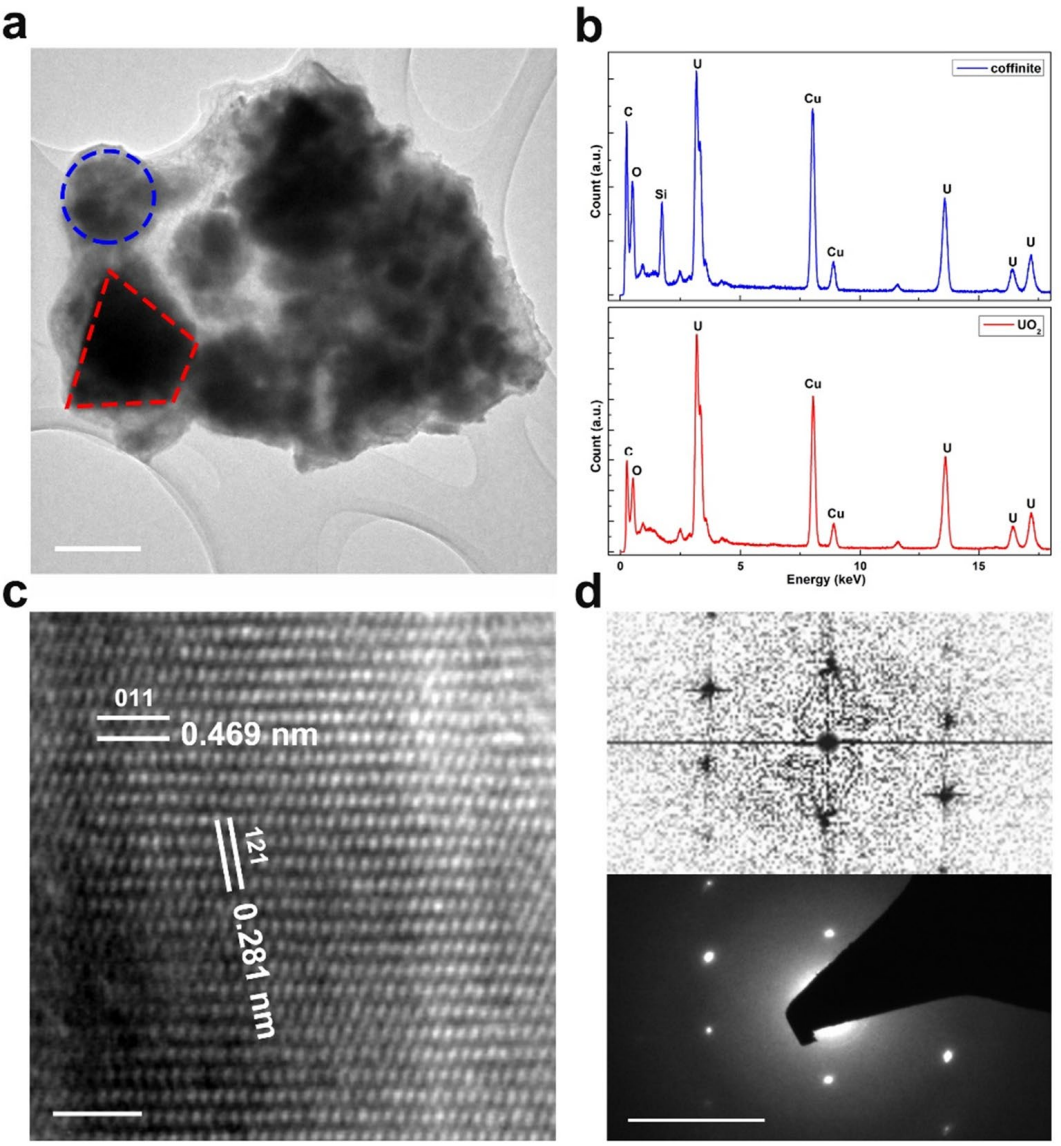
(**a**) TEM images of the sample after 339 days leaching. The blue dotted circle indicates a neoformed coffinite grain, while the red dotted quadrangle indicates a UO_2_ grain. Scale bar 200 nm. (**b**) EDX spectrum obtained from the coffinite and the UO_2_ grain, respectively. Carbon and Copper signals originate from the TEM grid with lacey carbon film. (**c**) High-resolution TEM image of the coffinite grain, as indicated by the blue circle in (**a**). Scale bar 2 nm. (**d**) Fast Fourier Transform of the HRTEM image in (**c**) and the diffraction pattern of this coffinite grain. Scale bar 21/nm.

In the TEM image (Fig. 6a), the morphology of the grains indicated by the blue dotted circle and the red dotted quadrangle differ. Additionally, in the Energy-Dispersive X-ray (EDX) spectrum obtained from the former, there appeared to be a strong signal from Si that did not appear in the spectrum from the latter (Fig. 6b). This suggests that the grain indicated by the blue circle may be a neoformed coffinite particle. To determine the structure of this particle, the high-resolution TEM image was analyzed (Fig. 6c). The d-spacings between the two sets of white lines were 0.469 nm and 0.281 nm, respectively, which were identified as the (011) and (121) in coffinite^14,18^. Neither of these two d-spacings belonged to UO_2_. The fast Fourier transform (FFT) of the image agreed well with the electron diffraction pattern of this grain, as shown in Fig. 6d. These results clearly indicated the formation of the neoformed coffinite.

## Conclusion

The first evidence of coffinite formation from UO_2_ has been obtained at low temperature under conditions relevant for geological disposal of SNF and in uranium ore deposits. In a solution slightly undersaturated with respect to SiO_2_(am) at 25 °C (i.e.[Si]_tot_ = (1.77 ± 0.03) × 10^–3^ mol L^−1^ and pH = 9), anoxic conditions provided Eh values in a range that caused the dissolution of UO_2_ through oxidative weathering, followed by precipitation of coffinite. In the studied conditions, the precipitation of coffinite was thermodynamically favored as compared with the mixture of UO_2_⋅2H_2_O (am) and SiO_2_ (am). Using current thermodynamic databases, geochemical calculations showed that coffinite coexisted with UO_2_(OH)_3_^−^ predominant species in solution. From a kinetic point of view, the formation of coffinite occurred rapidly and the precipitation of coffinite was unambiguously evidenced after 155 days of leaching at the surfaces of the UO_2_ pellet.

These results explained the common occurrence of coffinite in sedimentary uranium ore deposits and showed that coffinite should be considered in modeling the long-term behavior of SNF in a geologic repository. The formation of coffinite is a mechanism that reduces the amount of uranium released from the SNF, especially in the event of an increase of the redox potential of the groundwater. This mechanism may also reduce the release of tetravalent actinides, such as plutonium, which form solid solutions with tetragonal structure of coffinite^34,35^ but it could also trigger the release of other radionuclides which do not fit into the coffinite structure, as demonstrated by the release of radiogenic Pb occuring during coffinitization^3^.

## Experimental methods

### Preparation of UO_2_ pellet

The oxalic acid used to perform the synthesis was purchased from Sigma-Aldrich in analytical grade. The uranium (IV) chloride solution was prepared by dissolving metal chips provided by CETAMA (CEA France) in concentrated HCl solution (6 mol L^−1^). The final concentration of the stock solution was determined by Inductively Coupled Plasma-Atomic Emission Spectroscopy (ICP-AES) and was found to [U]_tot_ = 0.515 ± 0.001 mol L^−1^. The uranium oxalate precursor was obtained by direct precipitation in an opened vessel in application of the protocol reported by Hingant et al.^36^ The weighed oxalic acid was dissolved in about 50 mL of deionized water at 60 °C, then the uranium solution was added slowly leading to the direct precipitation of the oxalate precursor. The molar ratio of oxalic acid/uranium was equal to 3. The mixture was left under continuous stirring for 30 min, then centrifuged twice with deionized water and finally once with ethanol in order to eliminate the excess of oxalic acid. The resulting powder was dried overnight in an oven at 90 °C. The prepared oxalate was calcined at 600 °C for 6 h under Ar/H_2_ (5%) atmosphere in order to maintain uranium under its tetravalent oxidation state. The calcination at 600 °C was considered as a compromise to ensure the complete conversion of the powder with less residual carbon and to obtain a powder with a specific surface area that promoted the densification by sintering. The specific surface area (SSA) of the UO_2_ powder was analyzed using 10 points krypton adsorption isotherm and the B.E.T. method (ASAP 2020, Micromeritics). The SSA of the starting powder reached 8 m^2^ g^−1^.

UO_2_ pellet was prepared by uniaxial pressing at 500 MPa of 1.2 g of the obtained UO_2_ powder in a dye of 13 mm in diameter. The pellet was placed in a carbon furnace then sintered at 1,700 °C for 8 h under vacuum. The pellet obtained was then polished with successive grain size of 10 µm, 5 µm and 1 µm. Finally, a polishing step using colloidal silica was achieved to eliminate micro-scratches and to obtain optical grade polished surface. The pellet was then placed in an ethanol bath and sonicated in order to remove all traces of silica particles that could remain at the surface of the sample. The mass of the pellet after polishing was equal to 1.105 ± 0.001 g. The densification rate of the pellet was determined by combining geometric measurements thanks to a caliper splint and helium pycnometry. The apparent density of each pellet was evaluated by geometrical measurements and compared to the calculated density of UO_2_ (*d*_*calc*_ = 11 g.cm^-3^). Measurement of the effective density by helium pycnometry allowed the differentiation between the open and closed porosity. The densification rate of the pellet was 93 ± 1% (indicating 7% porosity of which 3% was determined to be closed).

The specific surface area of the pellet was too low to be measured using Kr adsorption. Thus, it was estimated from SEM images recorded at low magnification and He pycnometry. SEM images of 92 × 62 µm^2^ were binarized using the FiJi software to determine the surface area of the pores in each investigated domain. The pore diameter distribution was evaluated from these images using the “analyse particles” plugin implemented in the FiJi software. The height of the pores was calculated to meet the volume of open porosity deduced from apparent and effective densities. Then, the surface area associated to the pores was obtained assuming that the pore size distribution was representative of the whole sample and that the pores were cylindrical. The resulting surface was divided by the mass of the sample to evaluate the specific surface area. An average value of the specific surface area was deduced from the analysis of 5 images recorded at low magnification^37^. From SEM images analysis, the specific surface area of the pellet was estimated to be (1.6 ± 0.9) × 10^–3^ m^2^ g^−1^.

### Leaching experiment

Leaching test was performed under anoxic conditions at 25 °C by contacting the UO_2_ pellet with a solution slightly undersaturated with respect to amorphous silica but oversaturated with respect to USiO_4_ coffinite at pH = 9. With this aim, 500 mL of the leaching solution were first prepared by adding sodium metasilicate (Na_2_SiO_3_ Sigma-Aldrich, analytical grade) to deionized water. The deionized water used for the preparation of the solution was previously out-gassed by boiling it for 2 h then cooled under bubbling with N_2_. The leaching solution was then stored for several days in the Ar-flushed glove box before being used in order to reach equilibrium with the partial pressure of O_2_(g) in the glove box (pO_2_ ≤ 1 ppm). The silicate concentration in the solution analyzed by ICP-AES reached [Si]_tot_ = (1.77 ± 0.03) × 10^–3^ mol L^−1^ whereas the inorganic carbon (IC) content was found to [IC] = (1.6 ± 0.1) × 10^–4^ mol L^−1^ from measurement with TOC-meter apparatus (Shimadzu).

100 mL of the solution was introduced in a teflon container (Savillex) with the UO_2_ pellet. The solution was immediately spiked with 1.8 µL of the uranium stock solution in order to increase the uranium elemental concentration to 10^–5^ mol L^−1^. Then, the pH was adjusted to pH = 9 with the help of 8 mol L^−1^ of NaOH solution (Carlo Erba, ACS reagent).

During the experiment, the container was closed most of the time, except to monitor the pH and Eh values and to sample the solution. The pH was measured using a Mettler Toledo InLab Expert Pro-ISM electrode against pH buffers (Inlab Solutions, Mettler Toledo, pH = 2.00; 4.01, 7.00 and 9.21 at 25 °C). The redox potential of the solution was monitored using a Pt combined electrode (Mettler Toledo InLab Redox) stored in the glove box. According to the value of the potential vs. NHE of the Ag/AgCl, KCl (3 mol L^−1^) electrode, Eh was calculated as: Eh = E_meas_(Pt) + 207 mV. At regular time intervals, 1–4 mL of the solution were taken off, then centrifuged at 14,000 rpm for 5 min, acidified up to 2% using HNO_3_ (69% from VWR Chemicals) and stored at 4 °C before analysis. After 328 days of leaching, an additional centrifugation step was performed at 9,000 rpm for 20 min using ultra-filtration PES membrane of 3 KDa in Vivaspin tubes. The samples were analyzed either by ICP-AES (Spectro Arcos EOP) or by ICP-MS (Thermo Scientific iCAP RQ). The calibration was performed using PlasmaCAL (SCP Science) single element calibration standards ([U]_tot_ or [Si]_tot_ = 1,000 ppm) diluted in 1% HNO_3_ solution. Concentrations and associated uncertainties were respectively the average and twice the standard deviation of three replicates. For ICP-MS analysis of U elemental concentration, Bi and Ir were used as internal standards.

The pellet was removed several times from the leaching solution to perform Grazing Incidence XRD (GI-XRD) and Environmental Scanning Electron Microscope (ESEM) analyses. For that purpose, the pellet was softly rinsed with deionized water and gently dried on an absorbing towel, then it was introduced in airtight sample-holder sealed in the Ar glove box before analyses. The sample-holder was not opened during GI-XRD analysis to avoid long-term exposure to ambient atmosphere.

### Characterizations of UO_2_ pellet

The UO_2_ samples were characterized by powder X-ray diffraction (PXRD). PXRD patterns were recorded using a Bruker D8 advance diffractometer with copper radiation (λCu Kα_1,2_ = 1.54184 Å) in a parallel mode and using the reflection geometry. The patterns were recorded between 5° and 100° (2θ) with a step of 0.02° and a counting time of 3 h. The resulting data were refined using the Fullprof_suite^38^ by applying the Rietveld method and using the Thomson Cox profile function^39^. Pure silicon was used as a standard to determine instrumental parameters. Zero shift, unit cell parameters, overall displacement, preferred orientation and anisotropic size model for the microstructural characteristics were considered for all the refinements. The PXRD pattern of the prepared powder showed UO_2+x_ (fluorite structure-type, space group Fm $\overline{3}$ m) with lattice parameter: a = 546.77(1) ppm.

Grazing Incidence XRD (GI-XRD) patterns were recorded using a Bruker D8 Advance diffractometer equipped with a motorized reflectivity stage which allowed vertical translation of the sample. The complete primary optics setup was already described by Szenknect et al.^40,41^ and was composed of a Cu Kα_1,2_ (λ = 1.54184 Å) source, a Göbel mirror, a motorised divergence slit, a fixed 0.2 mm slit, an automatic absorber, a fixed 0.1 mm slit after the absorber, and 2.5° Sollers slits. The secondary optics included a motorised anti-scattering slit, a graphite monochromator, 2.5° Sollers slits, a 0.05 mm receiving slit and a point detector. GI-XRD diffractograms were obtained at θ_*i*_ = 1° to evidence the presence of coffinite phase at the pellet surface.

The microstructure of the prepared pellet was first observed using a Quanta 200 Environmental Scanning Electron Microscope (ESEM-FEG, FEI Company) equipped with a backscattered electron detector (BSED) in high vacuum conditions with a 8 kV accelerating voltage and a 7 mm working distance. During the leaching experiment, the surface of the pellet was also regularly observed under environmental conditions. According to Podor et al.^42^, the pellet was directly introduced in the ESEM chamber equipped with a Peltier stage without any further preparation. The Peltier stage was cooled down to 2 °C prior the introduction of the sample. A great caution was paid to the pumping sequence in order to avoid any dehydration of the sample. This pumping sequence consisted in 5 differential pumping steps between 50 and 200 Pa of water. Finally, the water vapor pressure in the chamber was adjusted to 40 Pa which corresponded to a relative humidity of 5.7%. With this experimental procedure, the sample was never dried. The use of the ESEM under wet conditions prevented the dissolution experiment from perturbations induced by the sample observation.

X-Ray Photoelectron Spectroscopy (XPS) analyses were carried out with Thermofisher Escalab 250 XI using a monochromatic Al Kα source (*hν* = 1,486.6 eV)^43^. Due to a charge effect the samples were analysed using a charge compensation flood gun. The instrument was calibrated to the silver Fermi level (0 eV) and to the 3*d*_*5/2*_ core level of metallic silver (368.3 eV). The C-*1s* signal for adventitious carbon was used to correct the charge effect. The C–C/C–H component of C-*1s* spectra was fixed at 285.0 eV. The analysis zone was 900 µm diameter spot. The pass energy for overview and high resolution spectra was 150 eV and 20 eV, respectively. The data processing was performed using the commercial Avantage software. For the fitting procedure, a Shirley background has been used.

Transmission Electron Microscopy (TEM) characterization were carried out using a FEI Tecnai G2 F20 X-TWIN Transmission Electron Microscope operated at 200 kV, equipped with an energy dispersive spectroscopy (EDS) system. Fast Fourier transformation (FFT) and image filtering were performed using DigitalMicrograph software. The samples after leaching were ultrasonicated in acetone for 30 min at room temperature to separate coffinite particles from the surface of the pellets. The solutions after ultrasonication were dripped onto TEM grids with lacey carbon thin film using pipettes. For each TEM sample, only thin area on the edge of each particle was observed for ideal high-resolution imaging conditions.

### Modeling of the U speciation in solution

Phreeqc^44^ and GibbsStudio^45^ codes were used to model the aqueous chemistry of the leaching experiment carried out with UO_2_ pellets. ThermoChimie Database vs 10^46,47^ was used with some modifications. Selected log K° values used for calculations are summarized in Table 1.

**Table 1:** Equilibrium constants of selected uranium phases and hydroxocomplexes.

Solubility	Log_10_*K°_s,0_	Reference
Coffinite + 4H^+ ⇆^ U^4+^ + H_4_SiO_4_	− 7.80	Coffinite (TC*)^48^
	− 5.25	Coffinite^22^
Coffinite (am) + 4H^+^^⇆^ U^4+^ + H_4_SiO_4_	− 1.5	Coffinite (am) Estimated from NEA guidelines
UO_2_⋅2H_2_O (am) + 4H^+^^⇆^ U^4+^ + 4H_2_O	1.5	^49^
Hydroxocomplexes	Log_10_*K°_(1,n)_	
U^4+^ + 3H_2_O + 3H_4_SiO_4_^⇆^ U(OH)_3_(H_3_SiO_4_)_3_^2−^ + 6H^+^	− 18.39	^15^

*TC stands for ThermoChimie Database.

Experimental data used for numerical modelling correspond to that obtained at equilibrium (> 100 days) after ultrafiltration of the samples.

## Supplementary information

Supplementary Information.
