# Supplementary material for: Coffinite formation from UO2+x
Source: Sci Rep. 2020 Jul 22;10:12168. doi: 10.1038/s41598-020-69161-1 (PMC7376029; doi:10.1038/s41598-020-69161-1)
Supplement: Supplementary file 1 — Supplementary Information. [file 41598_2020_69161_MOESM1_ESM.docx]

Coffinite formation from UO_2+x_

Stéphanie Szenknect^a,^*, Delhia Alby^a^, Marta López García^b^, Chenxu Wang^d^, Renaud Podor^a^, Frédéric Miserque^e^, Adel Mesbah^a^, Lara Duro^b^, Lena Zetterström Evins^c^, Nicolas Dacheux^a^, Jordi Bruno^b^, Rodney C. Ewing^d^

^a^ ICSM, Univ Montpellier, CEA, CNRS, ENSCM, 30207 Bagnols sur Cèze, France

^b^ Amphos 21, Consulting, Carrer Veneçuela, 103, Planta 2, 08019, Barcelona, Spain

^c^ Swedish Nuclear Fuel and Waste Management Co, Blekholmstorget 30, SE-101 24 Stockholm, Sweden

^d^ Department of Geological Sciences, Stanford University, Stanford, CA 94025-2115, USA

^e^ DES-Service de la Corrosion et du Comportement des matériaux dans leur Environnement (SCCME), CEA, Université Paris-Saclay, 91191 Gif-Sur-Yvette, France

**Supplementary data**





*Figure S1: Rietveld refinement of XRD data for UO_2_ powder (experimental, simulated data and experimental – simulated data).*





*Figure S2: Rietveld refinement of XRD data for UO_2_ pellet (experimental, simulated data and experimental – simulated data).*

*Figure S3. ESEM micrographs (BSE mode) of the UO_2_ pellet recorded at different leaching times and low magnification. The red circles are examples of area where grains detachment is observed. Scale bar 50 µm.*
